# Association Between Serum Levels of Carotenoids and Serum Asymmetric Dimethylarginine Levels in Japanese Subjects

**DOI:** 10.2188/jea.JE20130137

**Published:** 2014-05-05

**Authors:** Rika Watarai, Koji Suzuki, Naohiro Ichino, Keisuke Osakabe, Keiko Sugimoto, Hiroya Yamada, Takeshi Hamajima, Nobuyuki Hamajima, Takashi Inoue

**Affiliations:** 1 Clinical Laboratory Medicine, Fujita Health University Graduate School of Health Sciences, Toyoake, Aichi, Japan; 2 Department of Public Health, Fujita Health University School of Health Sciences, Toyoake, Aichi, Japan; 3 Department of Clinical Physiology, Fujita Health University School of Health Sciences, Toyoake, Aichi, Japan; 4 Department of Hygiene, Fujita Health University School of Medicine, Toyoake, Aichi, Japan; 5 Department of Healthcare Administration, Nagoya University Graduate School of Medicine, Nagoya, Japan

**Keywords:** asymmetric dimethylarginine, carotenoids, cross-sectional study

## Abstract

**Background:**

Asymmetric dimethylarginine (ADMA) is an endogenous inhibitor of endothelium nitric oxide synthase (NOS). ADMA binds to a substrate-binding site of NOS and then inhibits nitric oxide production from vascular endothelial cells. Elevated ADMA levels are a risk factor for cardiovascular disease. Recently, it was reported that plasma ADMA levels were negatively correlated with vegetable and fruit consumption. The purpose of this study was to examine the association between serum levels of carotenoids and serum ADMA levels in Japanese subjects.

**Methods:**

We conducted a cross-sectional study of 470 subjects (203 men and 267 women) who attended a health examination in August 2011. Serum levels of several carotenoids were separately measured by high-performance liquid chromatography. Serum ADMA levels were determined by using an enzyme-linked immunosorbent assay kit.

**Results:**

In women, the multivariate-adjusted odds ratios (ORs) of elevated serum ADMA levels were significantly decreased in the highest tertile for β-cryptoxanthin (OR 0.47, 95% CI 0.23–0.95), α-carotene (OR 0.39, 95% CI 0.18–0.79), and β-carotene (OR 0.36, 95% CI 0.17–0.73) compared to the lowest tertile. In men, significantly decreased ORs were observed in the highest tertiles of serum zeaxanthin/lutein (OR 0.23, 95% CI 0.06–0.69) and α-carotene (OR 0.26, 95% CI 0.07–0.82), and in the middle and the highest tertiles of serum β-carotene (OR 0.27, 95% CI 0.09–0.74 and OR 0.20, 95% CI 0.03–0.88, respectively) when the tertile cutoff points of women were extrapolated to men.

**Conclusions:**

Higher serum levels of carotenoids, such as α-carotene and β-carotene, may help to prevent elevated serum ADMA levels in Japanese subjects.

## INTRODUCTION

Nitric oxide (NO) is synthesized from L-arginine by endothelium NO synthase (eNOS) in the vascular endothelium. Endothelium-derived NO is involved in anti-atherosclerotic biological activities, including vasodilation, leukocyte adhesion restraint, inhibition of vascular smooth muscle cell proliferation, and platelet aggregation inhibitory action.^1^ Dysfunction of the endothelial L-arginine/NO pathway is associated with hypertension,^2^ coronary heart disease,^3^^,^^4^ and diabetes.^5^

Asymmetric dimethylarginine (ADMA) is an endogenous inhibitor of eNOS that competitively inhibits L-arginine oxidation. ADMA is combined in a substrate-binding site of NOS, but ADMA is not decomposed into NO. As a result, ADMA inhibits the NO production of the vascular endothelium. ADMA is a risk factor for endothelial dysfunction. Circulating plasma levels of ADMA are increased in patients with renal failure,^6^ hypertension,^7^^,^^8^ hyperlipidemia,^9^ type 2 diabetes,^10^ and coronary artery disease.^11^

ADMA formation occurs during the hydrolysis of methylated proteins and is catalyzed by protein arginine methyltransferases. ADMA is secreted into body fluids and excreted into the urine.^12^^,^^13^ Intracellular ADMA is hydrolyzed to dimethylamine and L-citrulline by dimethylarginine dimethylaminohydrolase (DDAH). The enzyme activity of DDAH is decreased by increased oxidative stress.

Plasma ADMA is negatively correlated with vegetable consumption in older healthy subjects.^14^ This association may play a role in the inverse association between vegetable consumption and cardiovascular disease (CVD) risk. However, the mechanisms underlying the association between vegetable consumption and ADMA are not completely understood.

Carotenoids are phytonutrients that have antioxidant properties.^15^^,^^16^ Circulating carotenoid concentrations are useful biomarkers of vegetable and fruit intake.^17^ Some epidemiological studies have reported that high circulating carotenoid levels were inversely related to the risk of CVD.^18^^,^^19^ We suggest that circulating carotenoids may play an important role in the inverse association of vegetable consumption with ADMA, because oxidative stress influences the metabolism of ADMA. To our knowledge, however, no epidemiological study has investigated the association between serum carotenoid levels and serum ADMA levels.

Here, we conducted the present study to determine the relationship between serum carotenoid levels and serum ADMA levels in Japanese subjects.

## METHODS

This cross-sectional study is part of the ongoing Yakumo study, a population-based prospective study of lifestyle-related disease conducted in Yakumo-cho, Hokkaido, Japan. The study enrolled 526 subjects (210 men and 316 women), aged 40–91 years, who attended a health examination held in Yakumo-cho in August 2011. We excluded 12 subjects who did not provide informed consent for the present study and 44 subjects for whom adequate serum samples were not available to measure serum ADMA levels. The remaining 470 eligible subjects (203 men and 267 women) were included in the analysis. This study was approved by the Ethics Committee of Fujita Health University (approval number 11-101).

Fasting blood samples were taken during the health examination, and sera were immediately separated from blood samples by centrifugation. Sera were stored in a refrigerator for up to three days during the administration of health examinations and were transported to our laboratory at −15°C after the completion of health examinations. Samples were then stored in a deep freezer at −80°C in our laboratory for up to six months. Serum levels of five carotenoids (zeaxanthin/lutein, β-cryptoxanthin, lycopene, α-carotene, β-carotene) were measured by high-performance liquid chromatography. We measured serum ADMA levels by using an enzyme-linked immunosorbent assay kit (ADMA Xpress ELISA kit; Immundiagnostik AG, Bensheim, Germany). The coefficients of variation (CVs) for the intra- and inter-assay of carotenoids were 4.5% to 9.2% and 9.2% to 20.0%, respectively.^20^ The intra- and inter-assay CVs for the measurement of ADMA were 5.8% (serum ADMA levels: 0.48 µmol/l) to 7.9% (0.19 µmol/l) and 7.6% (0.47 µmol/l) to 10.8% (0.19 µmol/l), respectively, values which were reported in the instruction manual as typical variation for the assay. Serum creatinine levels were measured using an enzymatic method. Other biochemical analyses of sera were performed using an auto-analyzer (JCM-BM9130; Nihon Denshi Co., Ltd., Tokyo, Japan) at the laboratory of Yakumo General Hospital on the day of the health examination. We calculated the creatinine-based estimated glomerular filtration rate (eGFR) [eGFR = 194 × creatinine^−1.094^ × age^−0.287^ (× 0.739 in women)] as a marker for renal function. Blood pressure, body height, and weight were measured during the health examination. The body mass index (BMI) was calculated as body weight (kg) divided by height (m) squared. The value for glycated hemoglobin (HbA1c; %) was estimated as a National Glycohemoglobin Standardization Program (NGSP) equivalent value (%) calculated by the formula; HbA1c (NGSP) = 1.02 × HbA1c (JDS) + 0.25%, considering the relational expression of HbA1c (JDS) (%) measured by the previous Japanese standard substance and measurement methods and HbA1c (NGSP).^21^

Trained nurses obtained health information, including smoking habits (current smoker, former smoker, or never smoker), alcohol consumption (regular drinker, former drinker, or never drinker) and history of major illness (yes or no), using a questionnaire regarding health and daily lifestyle habits.

Statistical analyses were performed with the statistical software JMP ver. 10 (SAS Institute Inc., Cary, NC, USA). Since serum levels of carotenoids and ADMA were distributed logarithmically, we used log-transformed values for the analyses. The analysis of covariance and Tukey-Kramer HSD tests were conducted by sex to compare continuous parameters among quartiles of serum ADMA or tertiles of serum carotenoids. We analyzed sex differences in continuous parameters using Student’s *t*-test. The chi-square test was used to compare categorical variables. Serum levels of carotenoids and ADMA were represented as geometric means and 25th–75th percentile ranges. Other variables were represented as mean ± standard deviation (SD). Adjusted odds ratios (ORs) with 95% confidence intervals (CI) were estimated by multivariate logistic regression analysis. We divided the distribution of the serum carotenoid levels into tertiles and calculated the odds of elevated serum ADMA levels (greater than the 75th percentile) among tertiles of serum carotenoid levels using the lowest tertile as a reference. In logistic regression analysis, we included variables that might confound the association of serum carotenoid levels with serum ADMA levels, including age, smoking habits, drinking habits, BMI, eGFR, serum γ-glutamyl transpeptidase (γ-GTP) activities, hypertension, diabetes, and dyslipidemia. Because it has been reported that liver function affects serum levels of ADMA and carotenoids,^22^^,^^23^ γ-GTP activities were included in analysis along with other potential confounders. Hypertension was defined as systolic blood pressure greater than 140 mm Hg and/or diastolic blood pressure greater than 90 mm Hg (based on guidelines from the Japanese Society of Hypertension)^24^ and/or having a history of hypertension. Diabetes was defined as fasting plasma glucose level of 126 mg/dL or higher and/or HbA1c value of 6.5% or higher and/or a history of diabetes. Dyslipidemia was defined as a high-density lipoprotein cholesterol (HDL-C) level of less than 40 mg/dL, and/or a low-density lipoprotein cholesterol (LDL-C) level of 140 mg/dL, and/or a triglyceride level of 150 mg/dL or higher (based on the guidelines of the Japan Atherosclerosis Society),^25^ and/or a history of dyslipidemia. Moreover, we calculated ORs and 95% CIs for elevated serum ADMA levels in men using the tertiles cutoff points of women, to determine if sex differences in optimum cutoff points affect the results. All *P*-values for statistical analyses were two-tailed and values of <0.05 were considered statistically significant.

## RESULTS

Table 1 shows the characteristics of subjects stratified by sex and tertile of ADMA (<25th percentile, 25th–75th percentile, and ≥75th percentile). The mean age ± SD of study subjects was 66.5 ± 10.0 years for men and 65.6 ± 9.7 years for women. Serum ADMA levels were significantly higher in men than in women (*P* < 0.001). Serum levels of all carotenoids were significantly higher in women than in men (*P* < 0.001). Men had significantly higher serum levels of triglycerides and γ-GTP and significantly lower serum total cholesterol and HDL-cholesterol compared to women. Proportions of current smokers and current drinkers were significantly higher in men than in women. Serum γ-GTP activities decreased with increasing serum ADMA levels in men. In men, DBP was significantly lower in the middle group compared to that in the lowest quartile. The mean age was significantly higher in the middle group than in the lowest quartile in women.

**Table 1.** Characteristics of study subjects

		Men				Women				Sex differences
	
Percentiles of ADMA		≤25th	25th<, <75th	≥75th	Total	≤25th	25th<, <75th	≥75th	Total	*P*
*n*		51	101	51	203	67	133	67	267	
Age^a^	(years)	64.1 ± 9.9	67.8 ± 9.6	66.3 ± 10.7	66.5 ± 10.0	62.5 ± 10.4	67.0 ± 9.4^g^	65.7 ± 8.9	65.6 ± 9.7	0.319^d^
ADMA^b^	(µmol/L)	0.53 (0.50–0.56)	0.70 (0.64–0.77)^h,i^	1.02 (0.90–1.05)^h^	0.72 (0.58–0.86)	0.51 (0.48–0.55)	0.64 (0.60–0.68)^h,i^	0.81 (0.75–0.86)^h^	0.64 (0.56–0.71)	<0.001^d^
Zeaxanthin/lutein^b^	(µmol/L)	1.25 (0.93–1.77)	1.21 (0.87–1.84)	1.11 (0.87–1.47)	1.19 (0.90–1.72)	1.48 (1.23–2.06)	1.48 (1.20–2.02)	1.48 (1.23–2.01)	1.46 (1.20–1.97)	<0.001^d^
β-Cryptoxanthin^b^	(µmol/L)	0.17 (0.10–0.24)	0.19 (0.12–0.28)	0.18 (0.12–0.26)	0.18 (0.12–0.26)	0.34 (0.25–0.47)	0.31 (0.22–0.47)	0.29 (0.20–0.39)	0.31 (0.22–0.45)	<0.001^d^
Lycopene^b^	(µmol/L)	0.44 (0.25–0.82)	0.51 (0.32–0.79)	0.54 (0.35–0.82)	0.50 (0.33–0.82)	0.73 (0.52–1.09)	0.68 (0.45–1.08)	0.64 (0.40–1.14)	0.71 (0.48–1.10)	<0.0001^d^
α-Carotene^b^	(µmol/L)	0.17 (0.10–0.28)	0.20 (0.11–0.32)	0.16 (0.11–0.22)	0.18 (0.11–0.28)	0.31 (0.21–0.46)	0.27 (0.17–0.45)	0.23 (0.14–0.34)	0.27 (0.18–0.43)	<0.001^d^
β-Carotene^b^	(µmol/L)	0.55 (0.28–1.12)	0.73 (0.43–1.43)	0.57 (0.39–0.96)	0.64 (0.35–1.19)	1.39 (0.96–2.28)	1.41 (1.00–2.15)	1.16 (0.79–1.67)	1.35 (0.94–2.11)	<0.0001^d^
BMI^a^	(kg/m^2^)	24.1 ± 3.3	23.9 ± 3.0	24.2 ± 2.9	24.0 ± 3.0	23.4 ± 3.3	23.7 ± 3.6	23.4 ± 3.8	23.6 ± 3.6	0.141^d^
SBP^a^	(mm Hg)	136.8 ± 19.7	131.0 ± 15.6	133.3 ± 15.8	133.0 ± 16.9	131.8 ± 15.2	135.0 ± 18.9	133.9 ± 16.4	133.9 ± 17.4	0.585^d^
DBP^a^	(mm Hg)	82.8 ± 12.1	78.1 ± 10.2^f^	78.6 ± 9.7	79.4 ± 10.7	78.7 ± 8.8	78.3 ± 9.0	78.5 ± 7.6	78.5 ± 8.6	0.285^d^
Triglycerides^b^	(mg/dL)	114.6 (80.0–155.0)	100.5 (76.0–133.0)	110.1 (84.0–142.0)	106.3 (78.0–141.0)	85.9 (64.0–120.0)	86.8 (65.0–115.0)	98.2 (72.0–119.0)	89.3 (67.0–117.0)	<0.001^d^
Total cholesterol^a^	(mg/dL)	213.4 ± 32.1	211.3 ± 31.1	204.2 ± 34.6	210.0 ± 32.3	223.7 ± 34.7	221.0 ± 35.4	226.5 ± 34.9	223.0 ± 35.0	<0.0001^d^
HDL-cholesterol^a^	(mg/dL)	56.7 ± 12.0	56.6 ± 13.3	53.3 ± 12.3	55.8 ± 12.7	66.0 ± 14.0	64.4 ± 12.9	64.9 ± 14.5	64.9 ± 13.6	<0.001^d^
LDL-cholesterol^a^	(mg/dL)	121.9 ± 30.5	123.6 ± 28.7	120.4 ± 31.8	122.4 ± 29.8	126.5 ± 33.4	126.8 ± 31.3	130.3 ± 30.0	127.6 ± 31.4	0.068^d^
HbA1c^a^	(%)	5.6 ± 0.7	5.6 ± 0.5	5.6 ± 0.5	5.6 ± 0.6	5.6 ± 0.6	5.6 ± 0.6	5.5 ± 0.4	5.6 ± 0.6	0.571^d^
γ-GTP^b^	(IU/L)	50.8 (27.0–94.0)	37.0 (24.0–53.5)^f^	35.8 (23.0–47.0)^f^	39.7 (24.0–62.0)	25.3 (14.0–29.3)	24.1 (15.0–38.0)	22.3 (14.0–39.0)	23.9 (15.0–35.0)	<0.001^d^
eGFR^a^	(mL/min/1.73 m^2^)	71.4 ± 13.7	69.0 ± 15.7	75.4 ± 16.8	71.2 ± 15.7	71.2 ± 13.6	71.4 ± 15.4	68.3 ± 13.1	70.6 ± 14.4	0.657
Hypertension^c^		37 (72.6)	55 (54.5)	27 (52.9)	119 (58.6)	33 (49.3)	87 (65.4)	40 (59.7)	160 (59.9)	0.776^e^
Diabetes^c^		8 (15.7)	17 (16.8)	5 (9.8)	30 (14.8)	7 (10.5)	15 (11.4)	5 (7.5)	27 (10.2)	0.129^e^
Dyslipidemia^c^		30 (58.8)	48 (48.0)	27 (52.9)	105 (52.0)	35 (52.2)	64 (48.5)	39 (58.2)	138 (51.9)	0.983^e^
Smoking habit^c^	Never	15 (29.4)	26 (25.7)	10 (19.6)	51 (25.1)	57 (85.1)	110 (82.7)	54 (80.6)	221 (82.8)	<0.001^e^
	Former	26 (51.0)	59 (58.4)	32 (62.8)	117 (57.6)	6 (9.0)	13 (9.8)	9 (13.4)	28 (10.5)	
	Current	10 (19.6)	16 (15.8)	9 (17.7)	35 (17.2)	4 (6.0)	10 (7.5)	4 (6.0)	18 (6.7)	
Drinking habit^c^	Never	10 (19.6)	29 (28.7)	22 (43.1)	61 (30.1)	51 (76.1)	98 (73.7)	46 (68.7)	195 (73.0)	<0.001^e^
	Former	1 (2.0)	10 (9.9)	1 (2.0)	12 (5.9)	1 (1.5)	2 (1.5)	2 (3.0)	5 (1.9)	
	Current	40 (78.4)	62 (61.4)	28 (54.9)	130 (64.0)	15 (22.4)	33 (24.8)	19 (28.4)	67 (25.1)	

Abbreviations: ADMA, asymmetric dimethylarginine; BMI, body mass index; SBP, systolic blood pressure; DBP, diastolic blood pressure; eGFR, estimated glomerular filtration rate; HDL, high-density lipoprotein; LDL, low-density lipoprotein; HbA1c, hemoglobin A1c; γ-GTP, γ-glutamyltranspeptidase.

^a^Data are expressed as mean values ± standard deviation.

^b^Data are expressed as geometric mean values and 25th–75th percentiles in parentheses.

^c^Data are expressed as number and percentages in parentheses.

^d^*t* test (203 men vs 267 women).

^e^Chi-squared test (203 men vs 267 women).

^f^*P* < 0.05 (vs lowest quartile, Tukey-Kramer HSD test).

^g^*P* < 0.01 (vs lowest, Tukey-Kramer HSD test).

^h^*P* < 0.001 (vs lowest quartile, Tukey-Kramer HSD test).

^i^*P* < 0.001 (vs highest quartile, Tukey-Kramer HSD test).

Table 2 shows the serum levels of ADMA according to tertiles of serum carotenoid levels by sex. In women, serum ADMA levels were significantly decreased in the highest tertiles of serum α-carotene and β-carotene (*P* = 0.019, 0.035, respectively) compared to those in the lowest tertiles. There were no significant differences in serum ADMA levels between tertiles of serum carotenoid levels in men.

**Table 2.** Comparison of serum ADMA levels and serum carotenoid levels by sex

		Men		Women	
	
Percentiles of ADMA		*n*	ADMA (µmol/l)^a^	*n*	ADMA (µmol/l)^a^
Zeaxanthin/lutein	Low	68	0.73 (0.60–0.86)	89	0.63 (0.56–0.70)
	Middle	67	0.74 (0.59–0.90)	89	0.65 (0.57–0.73)
	High	68	0.69 (0.55–0.78)	89	0.64 (0.57–0.70)
	ANOVA *P*		0.240		0.391
β-Cryptoxanthin	Low	68	0.72 (0.58–0.86)	89	0.65 (0.58–0.75)
	Middle	67	0.72 (0.59–0.87)	89	0.64 (0.56–0.71)
	High	68	0.72 (0.60–0.86)	89	0.63 (0.56–0.69)
	ANOVA *P*		0.965		0.277
Lycopene	Low	68	0.72 (0.58–0.81)	89	0.65 (0.58–0.74)
	Middle	67	0.70 (0.56–0.86)	89	0.63 (0.55–0.69)
	High	68	0.74 (0.59–0.90)	89	0.64 (0.56–0.71)
	ANOVA *P*		0.580		0.527
α-Carotene	Low	68	0.72 (0.58–0.86)	89	0.67 (0.60–0.75)
	Middle	67	0.75 (0.61–0.92)	89	0.63 (0.56–0.73)
	High	68	0.69 (0.57–0.80)	89	0.62 (0.56–0.69)^b^
	ANOVA *P*		0.131		0.019
β-Carotene	Low	68	0.71 (0.57–0.86)	89	0.66 (0.58–0.77)
	Middle	67	0.75 (0.60–0.91)	89	0.64 (0.56–0.71)
	High	68	0.70 (0.61–0.81)	89	0.62 (0.56–0.68)^b^
	ANOVA *P*		0.300		0.035

Abbreviations: ADMA, asymmetric dimethylarginine.

^a^Data are expressed as geometric mean values, with the 25th–75th percentiles in parentheses.

^b^*P* < 0.05 (vs low, Tukey-Kramer HSD test).

Table 3 shows the age-adjusted and multivariable-adjusted ORs and 95% CIs for elevated serum ADMA levels of serum carotenoids by sex. In women, the age-adjusted OR for elevated serum ADMA levels were significantly decreased in the highest tertiles of serum β-cryptoxanthin (OR 0.47, 95% CI 0.23–0.95), α-carotene (OR 0.39, 95% CI 0.18–0.79) and β-carotene (OR 0.36, 95% CI 0.17–0.73) compared to the lowest tertile. Significant and inverse associations of serum β-cryptoxanthin, α-carotene, and β-carotene with elevated serum ADMA for women did not vary after adjusting for potential confounders. There were no significant associations between serum levels of carotenoids and elevated serum ADMA in men. In men, however, significantly decreased ORs were observed in the highest tertiles of serum zeaxanthin/lutein (OR 0.23, 95% CI 0.06–0.69) and α-carotene (OR 0.26, 95% CI 0.07–0.82), and in the middle and highest tertiles of serum β-carotene (OR 0.27, 95% CI 0.09–0.74 and OR 0.20, 95% CI 0.03–0.88, respectively) compared to the lowest tertiles as with women, when the tertiles cutoff points of women were extrapolated to men (Table 4). We calculated ORs and 95% CIs for elevated serum ADMA levels using the tertile cutoff points of men or women after the exclusion of current smokers, because smoking, a potent source of oxidative stress, may have a large effect on the results. Exclusion of smokers did not change the results (data not shown).

**Table 3.** Odds ratios for elevated serum ADMA levels^a^ in serum carotenoid tertiles by sex

Percentiles of ADMA		Men				Women			
	
		*n*	Range (µmol/L)	Age-adjusted Odds ratio (95%CI)	Multivariate adjusted Odds ratio (95%CI)^b^	*n*	Range (µmol/L)	Age-adjusted Odds ratio (95%CI)	Multivariate adjusted Odds ratio (95%CI)^b^
Zeaxanthin/lutein	Low	68	0.38–0.99	1	1	89	0.51–1.31	1	1
	Middle	67	1.00–1.49	1.67 (0.80–3.57)	1.75 (0.79–3.96)	89	1.32–1.82	1.12 (0.57–2.20)	1.05 (0.52–2.13)
	High	68	1.51–2.22	0.51 (0.21–1.22)	0.46 (0.17–1.17)	89	1.83–2.21	0.93 (0.46–1.87)	0.84 (0.41–1.75)
*P* for trend				0.170	0.159			0.835	0.650

β-Cryptoxanthin	Low	68	0.03–0.15	1	1	89	0.07–0.25	1	1
	Middle	67	0.16–0.24	1.03 (0.47–2.27)	0.87 (0.36–2.06)	89	0.26–0.39	0.75 (0.39–1.45)	0.74 (0.37–1.46)
	High	68	0.25–2.23	1.01 (0.46–2.25)	0.86 (0.35–2.07)	89	0.40–1.48	0.47 (0.23–0.95)	0.42 (0.20–0.88)
*P* for trend				0.975	0.739			0.038	0.023

Lycopene	Low	68	0.07–0.38	1	1	89	0.06–0.52	1	1
	Middle	67	0.39–0.70	1.44 (0.64–3.31)	1.55 (0.66–3.75)	89	0.53–0.99	0.65 (0.32–1.30)	0.61 (0.30–1.26)
	High	68	0.70–2.44	1.89 (0.86–4.28)	2.10 (0.91–5.01)	89	1.00–2.93	0.95 (0.49–1.85)	0.85 (0.42–1.70)
*P* for trend				0.115	0.085			0.882	0.652

α-Carotene	Low	68	0.02–0.13	1	1	89	0.05–0.20	1	1
	Middle	67	0.14–0.23	1.50 (0.70–3.25)	1.35 (0.60–3.06)	89	0.21–0.36	0.77 (0.40–1.46)	0.65 (0.32–1.29)
	High	68	0.24–2.15	0.65 (0.28–1.49)	0.51 (0.20–1.29)	89	0.37–1.90	0.39 (0.18–0.79)	0.30 (0.14–0.66)
*P* for trend				0.330	0.186			0.011	0.003

β-Carotene	Low	68	0.04–0.44	1	1	89	0.16–1.06	1	1
	Middle	67	0.45–0.98	1.55 (0.73–3.33)	1.28 (0.56–2.93)	89	1.07–1.88	0.68 (0.35–1.29)	0.60 (0.30–1.18)
	High	68	0.99–3.73	0.56 (0.23–1.35)	0.40 (0.14–1.09)	89	1.89–3.73	0.36 (0.17–0.73)	0.28 (0.12–0.60)
*P* for trend				0.234	0.093			0.006	0.001

Abbreviations: ADMA, asymmetric dimethylarginine; 95%CI, confidence intervals; BMI, body mass index; eGFR, estimated glomerular filtration rate; γ-GTP, γ-glutamyltranspeptidas.

^a^Elevated serum ADMA levels: greater than the 75th percentile of serum ADMA.

^b^Odds ratios adjusted for age, smoking habits, drinking habits, BMI, eGFR, serum γ-GTP activities, hypertension, diabetes and dyslipidemia.

**Table 4.** Odds ratios for elevated serum ADMA levels^a^ in men using the tertile cutoff points of women

Percentiles of ADMA		Range (µmol/L)	*n*	ADMA^b^ (µmol/l)	Age-adjusted Odds ratio (95%CI)	Multivariate adjusted Odds ratio (95%CI)^c^
Zeaxanthin/lutein	Low	0.38–1.31	121	0.73 (0.59–0.89)	1	1
	Middle	1.34–1.82	41	0.74 (0.57–0.88)	0.85 (0.37–1.86)	0.81 (0.33–1.90)
	High	1.83–2.22	41	0.66 (0.57–0.74)	0.25 (0.07–0.69)	0.23 (0.06–0.69)
*P* for trend				0.084	0.018	0.019

β-Cryptoxanthin	Low	0.03–0.25	142	0.71 (0.58–0.87)	1	1
	Middle	0.26–0.39	41	0.74 (0.62–0.87)	1.10 (0.48–2.40)	0.93 (0.38–2.15)
	High	0.41–2.23	20	0.70 (0.58–0.84)	0.74 (0.20–2.18)	0.72 (0.17–2.44)
*P* for trend				0.632	0.755	0.635

Lycopene	Low	0.07–0.51	99	0.71 (0.58–0.83)	1	1
	Middle	0.53–0.96	71	0.73 (0.59–0.90)	1.47 (0.73–2.95)	1.66 (0.79–3.52)
	High	1.01–2.44	33	0.73 (0.59–0.87)	1.12 (0.42–2.75)	1.20 (0.43–3.15)
*P* for trend				0.327	0.575	0.447

α-Carotene	Low	0.02–0.19	119	0.74 (0.59–0.89)	1	1
	Middle	0.20–0.35	54	0.68 (0.55–0.81)	0.59 (0.26–1.25)	0.56 (0.23–1.30)
	High	0.36–2.15	30	0.71 (0.62–0.80)	0.35 (0.10–0.99)	0.26 (0.07–0.82)
*P* for trend				0.131	0.037	0.021

β-Carotene	Low	0.04–1.06	145	0.73 (0.58–0.89)	1	1
	Middle	1.08–1.79	39	0.68 (0.56–0.79)	0.41 (0.15–1.02)	0.27 (0.09–0.74)
	High	1.91–3.73	19	0.70 (0.64–0.81)	0.27 (0.04–1.00)	0.20 (0.03–0.88)
*P* for trend				0.268	0.023	0.008

Abbreviations: ADMA, asymmetric dimethylarginine; 95%CI, confidence intervals; BMI, body mass index; eGFR, estimated glomerular filtration rate; γ-GTP, γ-glutamyltranspeptidase.

^a^Elevated serum ADMA levels: greater than the 75th percentile of serum ADMA.

^b^Data are expressed as geometric mean values and 25th–75th percentiles in parentheses.

^c^Odds ratios adjusted for age, smoking habits, drinking habits, BMI, eGFR, serum γ-GTP activities, hypertension, diabetes, and dyslipidemia.

## DISCUSSION

In this study, we found that serum levels of carotenoids, such as α-carotene and β-carotene, were negatively associated with serum ADMA levels in women. In men, there were significant negative associations between serum ADMA levels and serum carotenoids such as zeaxanthin/lutein, α-carotene, and β-carotene, when we extrapolated the tertile cutoff points of women to men. These associations were invariant even after adjusting for major confounders, including age, smoking and drinking habits, BMI, eGFR, serum γ-GTP activities, hypertension, diabetes, and dyslipidemia. Recently, Goralcyk et al^14^ reported that plasma ADMA levels were negatively associated with vegetable consumption. Our findings among women are consistent with the suggestion that carotenoids may play a role in the mechanism underlying the negative association between circulating ADMA levels and vegetable consumption.

ADMA inhibits endogenous NOS and reduces NO production. ADMA is a risk factor for endothelial dysfunction, and an elevated circulating ADMA level is an independent risk factor for progression of CVD^26^ and arteriosclerosis.^27^ Therefore, the inhibition of circulating ADMA may protect against CVD.

Urinary excretion removes a small amount of circulating ADMA, but the bulk of ADMA is degraded by DDAH after uptake from the circulation.^12^^,^^13^ DDAH is a hydrolytic enzyme for the clearance of ADMA and plays an important role in adjusting ADMA levels. Decreased DDAH activity may be the main mechanism responsible for increased ADMA levels. DDAH activity is decreased by increased oxidative stress. Because DDAH has a sulfhydryl group in its active catalytic site, DDAH is vulnerable to oxidative stress.^28^^,^^29^ Oxidative stress is caused by an imbalance between reactive oxygen species (ROS) generation and the antioxidant defense system. Increased oxidative stress also indicates enhanced production of ROS. Therefore, inhibition of oxidative stress by antioxidants may increase DDAH activity and prevent the increase of ADMA levels.

Carotenoids have antioxidant properties^16^ and anti-inflammatory effects.^30^ Serum carotenoid levels have been used as biomarkers of vegetable and fruit intake.^17^ Some epidemiological studies have shown that higher intake of vegetables and fruits^31^ or higher circulating carotenoid levels were associated with decreased CVD risk.^32^ The antioxidant properties and anti-inflammatory effects of carotenoids might explain the possible role of carotenoids in the prevention of CVD. This relationship may be one reason why increased vegetable consumption decreases CVD risk. However, it is uncertain whether circulating carotenoids increase the function of DDAH *in vivo*. Suppression of oxidative stress by carotenoids may stimulate DDAH activity, and the increase in circulating ADMA levels resulting from oxidative stress may be suppressed. This mechanism might play a role in the negative association between circulating carotenoid levels and CVD risk.

There was no significant association between serum levels of carotenoids and elevated serum ADMA in men in the present study. However, when the tertile cutoff points of women were extrapolated to men, significant associations between serum ADMA levels and serum levels of several carotenoids were observed in men as with women. Serum carotenoid levels were lower in men than in women. Fewer male subjects than female subjects had high enough serum carotenoid levels to decrease serum ADMA levels. Sex is well known to influence oxidative stress status.^33^ Oxidative stress was higher in men than in women because of the antioxidant properties of estrogen and the differences in muscle mass. Smoking and alcohol drinking, which were both more common among men in the present study, are associated with decreased serum carotenoid levels by increased oxidative stress^34^ and may have had an impact on the sex difference of oxidative stress.

We do not necessarily recommend dietary supplements, such as β-carotene, as large randomized trials have found that β-carotene supplementation is not effective for preventing CVD.^35^^,^^36^ Since high doses of carotenoids could have a pro-oxidant effect, one possibility is that the protective effects of carotenoids against CVD disappear at the high doses used in supplementation studies. We were unable to assess the effect of supplementation with β-carotene in our study, because only one man and three women in this study used β-carotene supplements.

The present study has several limitations. First, this study was unable to examine issues of causality because of the cross-sectional design. We hypothesized that increased serum levels of carotenoids may protect against decreased DDAH activity caused by oxidative stress. However, it is possible that the negative associations between serum ADMA levels and serum carotenoid levels are due to oxidative stress that is generated in the arteriosclerosis process, including endothelial dysfunction. A prospective study is thus required to confirm our results and clarify true causal relationships. Second, the statistical power of our analysis may not be high, especially for men, due to the small sample size. Lack of an association observed in analysis of men may mean that the sample size was not large enough to detect a weak association. Future larger studies are required to confirm our findings. Third, although confounding was appropriately adjusted for in our analysis, residual confounding cannot be completely ruled out.

In conclusion, higher serum carotenoid levels, such as α-carotene and β-carotene, were significantly and independently associated with decreased serum ADMA levels in a Japanese population. These findings suggest that maintaining a diet rich in vegetables and fruits could help prevent elevated ADMA levels, which are associated with the risks of arteriosclerosis and CVD.
